# Childhood Adversity Across the Lifespan: Assessing the Relationship Between ACEs and Subjective Cognitive Decline

**DOI:** 10.1093/geroni/igab046.548

**Published:** 2021-12-17

**Authors:** Elizabeth Avent, Jeanine Yonashiro-Cho, Roberta Peterson, Laura Mosqueda, Zachary Gassoumis

**Affiliations:** 1 University of Southern California, Los Angeles, California, United States; 2 Keck School of Medicine of USC, Los Angeles, California, United States; 3 Keck School of Medicine of USC, Alhambra, California, United States

## Abstract

Adverse childhood experiences (ACEs) have long-term impacts on health throughout the life course. Emerging research found that 3+ ACEs are associated with increased risk of cognitive impairment, nearly 11 times more than those who have not experienced childhood adversity. This study further investigates the ACEs-SCD relationship using data from the 2011 Behavioral Risk Factor Surveillance System (BRFSS). Seven ACE questions were asked of respondents in California, Washington, and Wisconsin (n=5,898, aged 55+); SCD was measured as experiencing progressive confusion or memory loss in the last 12 months. A series of logistic regressions were run to separately model the presence of ACEs and ACE score on SCD. Fourteen percent reported SCD, with 65.4% of those reporting 1+ ACE. More SCD respondents reported 4+ ACEs (10.8%) than non-SCD respondents (4.8%). The most frequently reported ACEs among those with SCD were psychological abuse (34.9%) and substance abuse in the household (30.5%). Regression results showed greater SCD risk with increased ACE scores, up to 2.90 odds of SCD for 4+ ACEs compared to 0 ACEs (p<.0001). Those reporting physical abuse and sexual abuse had the greatest odds (1.75 & 1.70, p<.0001) of SCD. Findings demonstrate a strong association between childhood adversity and SCD, with physical and sexual abuse placing individuals at greatest risk. Results show possible pathways to which ACEs can lead to cognitive impairment. Findings implicate the importance of considering a lifespan perspective in childhood adversity and family violence work and the importance of considering early-life adversity when assessing risk for cognitive impairment.

